# Mercury-Resistant Bacteria Isolated from an Estuarine Ecosystem with Detoxification Potential

**DOI:** 10.3390/microorganisms12122631

**Published:** 2024-12-19

**Authors:** Marynes Quintero, Sol D. Zuluaga-Valencia, Lady Giselle Ríos-López, Olga Sánchez, Cesar A. Bernal, Niza Sepúlveda, Javier Gómez-León

**Affiliations:** 1 Marine Bioprospecting Line, Evaluation and Use of Marine and Coastal Resources Program–VAR, Marine and Coastal Research Institute–INVEMAR, Santa Marta 470006, Magdalena, Colombia; marynes.quintero@invemar.org.co (M.Q.); sol.zuluaga@invemar.org.co (S.D.Z.-V.); lady.rios@invemar.org.co (L.G.R.-L.); 2 Department of Genetics and Microbiology, Faculty of Biosciences, Universitat Autònoma de Barcelona, 08193 Bellaterra, Spain; olga.sanchez@uab.cat; 3 Marine Environmental Quality Laboratory Unit–LABCAM, Marine Environment Quality Program–CAM, Marine and Coastal Research Institute–INVEMAR, Santa Marta 470006, Magdalena, Colombia; cesar.bernal@invemar.org.co; 4 Environmental Biotechnology Research Group, Faculty of Engineering, Technological University of Choco “Diego Luis Cordoba”, Quibdó 270001, Chocó, Colombia; nizasepulveda@gmail.com

**Keywords:** mercury detoxification, volatilization, *Stenotrophomonas* sp.

## Abstract

Mercury pollution is a significant environmental issue, primarily resulting from industrial activities, including gold mining extraction. In this study, 333 microorganisms were tested in increasing mercury concentrations, where 158 bacteria and 14 fungi were able to grow and remain viable at concentrations over 5.0 mg/L of mercuric chloride (II). One of the bacterial strains, *Stenotrophomonas* sp. INV PRT0231, isolated from the mouth of the San Juan River in the Chocó region in Colombia, showed a high mercury resistance level (MIC_90_ of 27 ± 9 mg/L), with a removal rate of 86.9%, an absorption rate of 1.2%, and a volatilization rate of 85.7% at pH 6.0 and 30.0 °C. The FTIR analysis showed changes in the functional groups, including fatty acid chains and methyl groups, proteins, and lipopolysaccharides associated with the carboxylate group (COO^−^), suggesting an important role of these biomolecules and their associated functional groups as mechanisms employed by the bacterium for mercury detoxification. Our study contributes to the understanding of the mechanisms of mercury biotransformation in microbial environmental isolates to help develop bioremediation strategies to mitigate mercury pollution caused by anthropogenic activities.

## 1. Introduction

Mercury (Hg) is a highly toxic and persistent heavy metal due to its bioaccumulative nature, raising significant global concerns. Hg pollution is not restricted to a particular geographical location. It can be transported thousands of miles due to its high residence time in the atmosphere and is hence regarded as the most debated environmental problem [1]. Hg is used in different industrial processes, including the processing of thermometers, manometers, paints, the paper industry, textile factories, cosmetics, mining extraction, and others, causing its release as waste into the environment [2].

In the environment, Hg may be present in elemental (Hg^0^), inorganic (Hg^2+^), and organic forms (CH_3_Hg and C_2_H_5_Hg), where the inorganic form of mercury II (Hg^2+^) is the most predominant in tropical and marine estuarine sediments [3]. In the Chocó biogeographic region in Colombia, the main sources of Hg pollution in the rivers are gold mining activities and wastewater [4,5]. The forms mercury II and methylmercury are introduced at surface waters such as rivers, streams, and estuaries through surface runoff and leaching from upper levels of soil before being transported to marine ecosystems [6].

Mercuric chloride (II), being water soluble, is one of the most potent toxic salts of mercury [7]. Today, a major issue, and a considerable threat to humanity, is tackling heavy metal pollution, which has been increasing, causing significant damage to the environment and public health. Many physical, chemical, and biological methods have been used in the past few decades to remediate contaminated soil and wastewater [8]. In terms of efficiency, cost-effectiveness, and sustainability, biological methods are the most effective. The reduction of mercury II is considered a natural detoxification mechanism developed by bacteria. The first process of mercury resistance starts with the translocation of Hg^2+^ into the plasma membrane and inside the cell to activate enzymatic mechanisms to reduce the Hg^2+^ to Hg^0^ [9]. The Hg^0^ escapes into the atmosphere due to its low water solubility and high vapor pressure [6]. Mercury-resistant bacteria harbor multiple genes that encode proteins for Hg resistance and exhibit specific mechanisms, such as direct efflux pumps, as well as extracellular or intracellular sequestration of the mercurial compounds to overcome Hg toxicity [1]. Due to its high specific response and efficient regulation, the *mer* operon has become the most attractive mechanism of Hg resistance and has been widely studied over the last decades [10]. Many studies have suggested the use of marine bacteria for the bioremediation of harmful metals as they have an immense ability to conquer the continuous fluctuating patterns of pH, temperature, salinity, and other variable environmental factors. The population of mercury-resistant bacteria (MRB) consists of Gram-positive as well as Gram-negative groups that can easily grow in a polluted environment containing Hg and can convert this toxic substance to a relatively nontoxic form [11]. With the concepts of environmental sustainability and management in mind, the bio-mitigation of Hg-contaminated media is an emerging need, requiring the discovery of new indigenous microbes with intrinsic Hg-tolerant capacities and detoxifying efficiency [12]. Different microbial genera have been reported for their ability to remove mercury, including *Pseudomonas*, *Proteus*, *Vibrio*, *Alteromonas*, *Aeromonas*, *Xanthomonas*, and Enterobacteriaceae [13]. In a previous work [14], 338 microorganisms were isolated from estuarine sediments in the Chocó region of Colombia. Among these, 276 microorganisms were evaluated in a heavy metal tolerance assay, where 110 were able to grow in a solid culture media supplemented with cadmium (Cd), lead (Pb), and zinc (Zn). In this study, we utilized 338 previously isolated strains to evaluate their mercury tolerance, selecting the most promising microorganism for further Hg^2+^ detoxification assays. Our study has significant biotechnological applications, particularly in bioremediation. The potential of utilizing native microbes and their genetic and metabolic pathways offers a sustainable solution for mining wastewater treatment and the bioremediation of environments contaminated with mercury.

## 2. Materials and Methods

### 2.1. Culture of Microorganisms

The microorganisms were obtained from the microbial collection of the Marine Museum of Natural History of Colombia—“Makuriwa” of INVEMAR—and stored at −80 °C, which consisted of 338 microorganisms previously phenotypically characterized, comprising 272 bacteria and 67 fungi. The isolation of microorganisms from estuarine ecosystems has been described in a previous study [14]. For microbial reactivation, the cryovials were taken and placed for thawing in an incubator at 30.0 °C. Aliquots of 100 µL were spread over the surface of the same culture media used for the isolation. The plates were incubated at 30.0 °C until growth was observed, and the colonies were compared with the phenotypic characteristics of the initial isolate.

### 2.2. Mercury Resistance Assay

A broth microdilution assay was made for the mercury tolerance evaluation. To this end, stock solutions of LB broth [15] supplemented with mercury chloride (HgCl_2_) at the concentrations of 10, 20, 40, 60, and 100 mg/L were prepared. The mercury stock solution was sterilized using a 0.22 μm filter. The inoculum cultures of bacteria and yeasts were prepared previously in 10 mL of LB broth (final pH 7.0 ± 0.2) and incubated at 30.0 °C in the dark for 72 h. Then, 100 µL of the stock solution was added in 96-well microplates and mixed with 100 µL of microbial suspension. Thus, the final concentrations of HgCl_2_ were 5, 10, 20, 30, and 50 mg/L. Three growth controls were included: (i) LB broth with the microorganism without HgCl_2_, (ii) non-inoculated LB broth (abiotic control), and (iii) LB broth with HgCl_2_ (abiotic control). The microplates were incubated at 30.0 °C in the dark for 72 h.

After this time, 2.0 µL of metabolic indicator resazurin Sigma^®^ (St. Louis, MO, USA) (0.4 mg/mL) was added to each well in order to measure cell viability. The microplates were then additionally incubated at 30.0 °C in the dark for 2 h. The viability was determined by the resazurin reduction measured at an absorbance of 570 and 603 nm using a spectrophotometer Thermo Scientific™ Multiskan™ GO (Vantaa, HEL, Finland). The cell viability percentage was calculated using Equation (1) [16], in which values equal to or over 80% were considered as viable cells:

(1) $$Viability\;\left(\%\right)=\frac{A_{Lw}-\left(A_{Hw}\times R_{o}\right)for\;Treated\;wells}{A_{Lw}-\left(A_{Hw}\times R_{o}\right)for\;Control\;wells}\times 100$$

where:

*A_LW_* = Absorbance at lower wavelength minus the blank;

*A_HW_* = Absorbance at higher wavelength minus the blank.

$$Correction\;factor\left(R_{o}\right)=\frac{{ABS}_{570\;nm}of\;resazurin\;in\;LB\;broth-{ABS}_{570\;nm}of\;LB\;broth}{{ABS}_{603\;nm}of\;resazurin\;in\;LB\;broth-{ABS}_{603\;nm}of\;LB\;broth}$$

where ABS is the absorbance.

For the fungi strains, the mercury tolerance assessment was conducted by measuring radial growth over the surface of solid LB medium (final pH 7.0 ± 0.2) supplemented with 10, 50, 100, 200, and 250 mg of HgCl_2_/L; a growth control in LB agar without HgCl_2_ was included. Growth was calculated measuring the radial development of treatments compared with the growth of the untreated control.

### 2.3. Minimum Inhibitory Concentration Assays

Six bacteria that showed cell viability in the broth microdilution assay using HgCl_2_ were selected for further experiments to determine the minimal inhibitory concentration (MIC), corresponding to the lowest concentration of Hg^2+^ (prepared with HgCl_2_) where no growth was observed. For this purpose, a bacterial suspension was prepared in LB broth [15], and 100 µL of this cell solution was added to 96-well microplates which contained LB broth with 5, 10, 20, 30, 40, 50, 60, 70, 80, 90, and 100 mg Hg/L (final concentrations). A growth control in LB broth without metal was also included. The microplates were incubated at 30.0 °C in the dark for 72 h. Growth was measured by optical density (OD) at 600 nm using a Thermo Scientific™ Multiskan™ GO spectrophotometer. Non-linear regression with four parameter logistic curves was used for the estimation of MIC. The tolerance to HgCl_2_ was classified according to the MIC values modified from [17] as high (MIC ≥ 22.0 mg Hg/L), moderate (7.0 < MIC < 22.0 mg Hg/L), and low (MIC ≤ 7.0 mg Hg/L).

Three bacteria exhibiting the highest MIC values were selected for taxonomic identification and were subjected to bacterial growth measurements following exposure to mercury (Hg^2+^ prepared with HgCl_2_) in liquid culture medium.

### 2.4. Taxonomic Identification

The extracted genomic DNA of the bacteria exhibiting the highest mercury tolerance was utilized to perform the amplification by PCR of the 16S rRNA gene, using the universal primer sets 27F-1492R. The PCR-amplified products were subjected to sequencing at Macrogen (Seoul, Republic of Korea) using the Sanger/capillary method, reading both strands to ensure the reliability of the sequencing process.

The closest relatives from partial sequences of the 16S rRNA gene were identified by comparing them with sequences available in the Refseq_rna (Reference RNA sequences) database of the National Center for Biotechnology Information (NCBI) (https://www.ncbi.nlm.nih.gov/ accessed on 6 September 2024). Alignment was performed using MUSCLE (Multiple Sequence Comparison by Log-Expectation), and phylogenetic analysis was conducted using MEGA11 version 11.0.13 employing the maximum likelihood method with 1000 Bootstrap replicates. The analysis was based on the Kimura 2-parameter nucleotide evolution model (K2+G) with a Gamma distribution (G). The 16S rRNA gene sequences were deposited in GenBank database with the accession numbers PQ508387, PQ508388 and PQ508389.

### 2.5. Measurement of Bacterial Growth and Mercury Concentration in Liquid Medium

Three bacteria were selected according to the MIC value in HgCl_2_. Briefly, a bacterial pre-inoculum was adjusted in LB broth [15] at pH 7.0 ± 0.2 to an optical density (600 nm) of 0.4 using a Thermo Scientific™ Multiskan™ GO spectrophotometer. Aliquots of 2.4 mL were added to a flask of 250 mL containing 60 mL of liquid LB medium at pH 7.0. Three group experiments were established: (a) a bacterial liquid culture in LB supplemented with 5.9 ± 0.7 mg Hg/L (prepared with HgCl_2_), (b) a growth control of bacteria in LB without mercury, and (c) four flasks as abiotic controls of LB broth without cells supplemented with the same mercury concentration, with duplicates sampled at 0 and 96 h to observe a possible abiotic mercury reduction [18]. Flask cultures were incubated in the dark in orbital agitation at 140 rpm, 30.0 °C for four days. All experiments were carried out in triplicate. Different aliquots of 300 µL at several time-points (0, 12, 24, 48, 72, and 96 h) were taken to measure the optical density (OD_600nm_) with a spectrophotometer (Thermo Scientific™, Vantaa, HEL, Finland) and obtain the microbial growth curves using the Multiskan Go software version 4.1. The specific growth rates were calculated from the OD measurements by analyzing the regression slope between the natural logarithm of OD and time, during the exponential growth phase.

For mercury concentration determination, the cultures were centrifuged twice at 4000 rpm at 4.0 °C for 30 min, and the supernatant obtained was adjusted to pH ≤ 2.0 with 65% Nitric Acid Supelco^®^ (Darmstadt, HE, Germany) and stored in the dark at 4.0 °C. The samples were processed using the nitric acid–sulfuric acid digestion method and sodium borohydride. The total mercury quantification was evaluated via cold vapor–atomic absorption (CV–AAAS) in an AT Thermo S Series iCE-330 spectrometer, Waltham, MA, USA using a calibration curve. The data are presented as mean ± standard deviation (SD).

The bacterial strain with the highest mercury detoxification percentage was selected for subsequent studies under slightly acidic conditions (pH 6.0 ± 0.2) to maintain mercury stability during the detoxification process.

### 2.6. Mercury Reduction at pH 6.0

The bacterium *Stenotrophomonas* sp. INV PRT0231 was selected for a new assay to evaluate the ability of mercury reduction. Briefly, a cellular suspension was adjusted at 0.4 optical density at 600 nm using a Thermo Scientific™ Multiskan™ GO spectrophotometer. First, 1000 µL of the cellular suspension was added in 25 mL of LB broth [15] at pH 6.0 ± 0.2. For the confirmation of mercury reduction, four group experiments were established: (i) a bacterial liquid culture in LB broth supplemented with 5.9 ± 0.6 mg Hg/L; (ii) a growth control of bacteria in LB broth without mercury; (iii) abiotic controls (sampled at 0 and 48 h in triplicate) of LB broth without cells and supplemented with the same mercury concentration as negative controls; and (iv) a killed control that consisted of biomass adjusted to an OD_600nm_ of 0.4 and inactivated by sterilization at 15 psi for 15 min, which represented the dead biomass suspension in LB broth supplemented with the same mercury concentration in order to observe a possible metal reduction by a biosorption process [18]. The flasks were incubated at 30.0 °C at 140 rpm during 48 h. Different time-points were measured for determining the microbial growth curve at 0, 2, 8, 18, 24, 30, and 48 h. All experiments were carried out in triplicate. The samples were centrifuged twice at 4000 rpm for 30 min at 4.0 °C. The supernatant obtained was acidified with ultrapure nitric acid and stored at 4.0 °C for total mercury (Hg T) quantification using a Direct Mercury Analyzer (Dual-cell DMA 80, Milestone, Sorisole, Italy). The Hg T quantification was performed in the Marine Environmental Quality Laboratory Unit—LABCAM (accreditation to ISO 17025:2017 [19])—following US EPA method 7473. Additionally, the cellular biomass was washed two times with 0.8% saline solution to freeze-dry. The dry biomass was employed for FT-IR analysis and Hg T quantification. The mercury removal, absorption, and volatilization rates were calculated using Equations (2)–(4) [20]:

(2) $$q_{1}=\left(\frac{a_{1}-b_{1}}{a_{1}}\right)$$

(3) $$q_{2}=\frac{c_{1}}{a_{1}}$$

(4) $$q_{3}=q_{1}-q_{2}$$

where *q*_1_ is the removal of mercury from liquid culture; *a*_1_ is the initial liquid culture mercury concentration; *b*_1_ is the supernatant mercury concentration at different sampling times; *q*_2_ is the absorption rate; *c*_1_ is the mercury content of biomass; and *q*_3_ is the volatilization rate. The data are shown in terms of percentage (%).

### 2.7. Fourier-Transform Infrared (FT-IR) Analysis

FT-IR spectra analysis was performed at different times of a culture treated with and without mercury (Hg^2^⁺ prepared with HgCl_2_) to investigate the interaction between the functional groups (FGs) of biomolecules from *Stenotrophomonas* sp. INV PRT0231 and Hg. The samples were analyzed using a Fourier-transform infrared (FT-IR) spectrophotometer (SHIMADZU IRTracer-100, Kyoto, Japan) with a DLATS detector and equipped with a PIKE MIRacleTM ATR accessory. The dry biomass sample was pressed onto the diamond crystal, and the spectra were recorded from 400 to 4000 cm⁻^1^ with 64 scans and a resolution of 8 cm⁻^1^.

### 2.8. Statistical Analysis

The data are presented as average values with standard deviations. The data analysis was performed using the Software OriginPro version 24 (OriginLab Corporation, Northampton, MA, USA). The Kruskal–Wallis test was applied to compare more than two independent groups to assess differences in the mercury resistance recorded in microbial isolates. The minimum inhibitory concentration (MIC) was estimated using a non-linear regression with a four-parameter logistic model (4PL).

## 3. Results

### 3.1. Mercury Tolerance Assay

A total of 172 out of 333 microorganisms (52%) showed mercury resistance, where the bacteria (*n*= 158) and the fungi (*n*= 14), were able to grow in LB medium supplemented with several mercury concentrations. According to the different concentrations tested using the metabolic indicator resazurin, we found that 16% of isolates (51 bacteria and 2 fungi) showed cell viability in a range of 20.0–50.0 mg/L of HgCl_2_, while 36% of isolates (107 bacteria and 12 fungi) grew between 5.0–10.0 mg/L of HgCl_2_, and 33% of isolates (85 bacteria and 25 fungi) did not show viability in the lowest concentration evaluated (MIC < 5.0 mg/L of HgCl_2_). Additionally, 51 microorganisms (15%) out of 333 evaluated in this study did not grow after 72 h in the growth control and the LB broth with the HgCl_2_ concentrations tested. It is important to mention that 5 out of 338 microorganisms reactivated in this study did not show viability (no growth) after the reactivation of cryovials stored at −80 °C from the microbial collection of Marine Museum of Natural History of Colombia—“Makuriwa” of INVEMAR.

**Figure 1:**
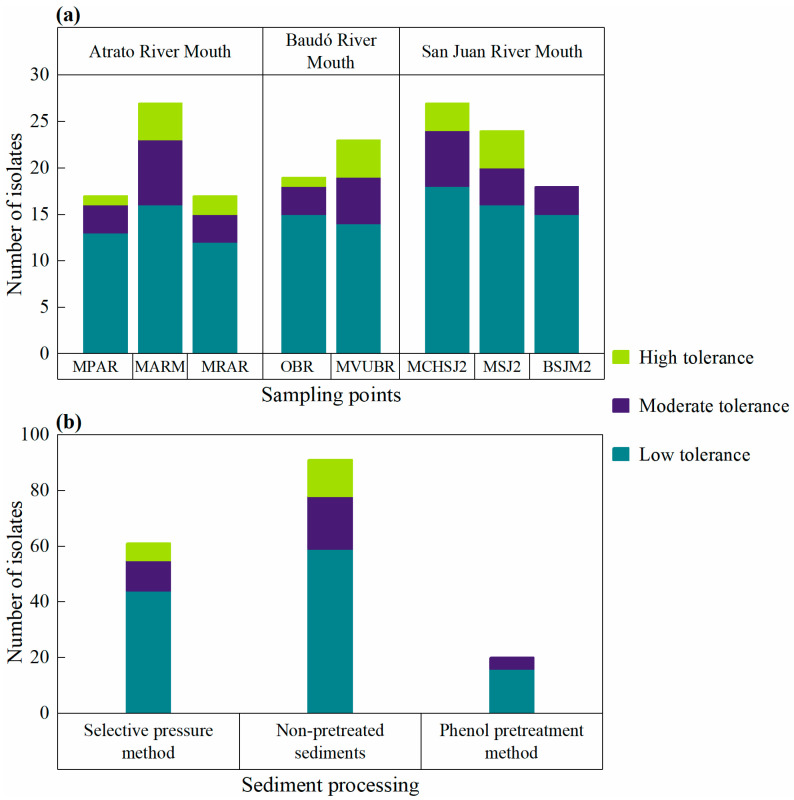
Number of mercury-resistant microorganisms isolated at the different sampling points (**a**) and using each processing method (**b**). Margarita Atrato River mouth (MARM), Mangrove Paila Atrato River (MPAR), Mangrove Roto Atrato River (MRAR), Mangrove Via Usaraga Baudó River (MVUBR), Out to Sea Baudó River (OBR), Mangrove Choncho San Juan River two (MCHSJ2), Mangrove San Juan River two (MSJ2), and Beach San Juan River mouth two (BSJM2).

The mercury-resistant tendency of microorganism isolates for the sampling points was San Juan River mouth > Atrato River mouth > Baudó River mouth, with values of 69, 61, and 42 microorganisms, respectively (Figure 1a). However, since only two sites were sampled in the locality of the Baudó River mouth (OBR and MVUBR), this could have resulted in a limited number of mercury-resistant microorganisms. On the other hand, the mercury resistance observed in the isolates obtained with the direct culture of non-pretreated sediments and the selective pressure method using mercury chloride was similar (*p* > 0.05), with values of 91 (53%) and 61 (35%) microorganisms, respectively (Figure 1b). In contrast, only 12% (*n* = 20) of the microorganisms isolated from the phenol pretreatment method showed mercury resistance.

Six bacterial strains, which showed the highest mercury resistance, were selected for further evaluation of growth in the LB medium [15] at increasing mercury concentrations (Hg^2+^ prepared with HgCl_2_). As shown in Figure S1, the highest MIC_90_ value (64 ± 19 mg Hg/L) was obtained for bacterium 76866 isolated from the Atrato River mouth; similarly, the strain 76980 isolated from the San Juan River mouth showed a high resistance, with an MIC_90_ value of 27 ± 9 mg Hg/L. On the other hand, three strains showed a moderate resistance to mercury, the strain 77020 isolated from the San Juan River mouth showed an MIC_90_ value of 6 ± 3 mg Hg/L, while the strains 77098 and 77137 isolated from the Baudó River mouth showed MIC_90_ values of 24 ± 11 mg Hg/L and 20 ± 4 mg Hg/L, respectively. Strain 77079 showed a low tolerance to mercury, with an MIC value lower than 3.0 mg Hg/L.

### 3.2. Taxonomic Identification of Selected Strains

**Figure 2:**
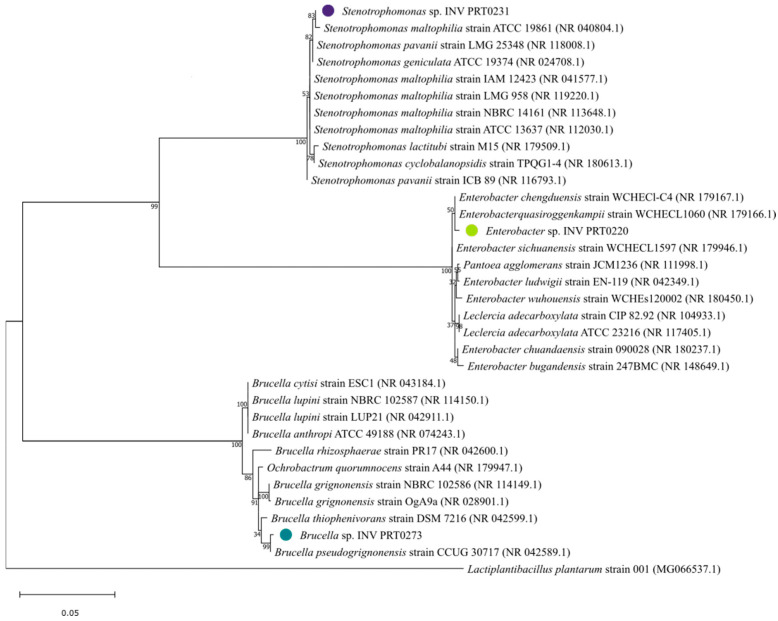
Phylogenetic tree of the three selected strains of mercury-resistant bacteria from this study (highlighted with a colored dot).

After the characterization of the MIC values of the previously selected strains, we finally chose three candidates (strains 76866, 76980, and 77137) which presented high and moderate MIC values for HgCl_2_ (20.0–50.0 mg/L). They were identified as *Enterobacter* sp. INV PRT0220 (strain 76866), *Stenotrophomonas* sp. INV PRT0231 (strain 76980), and *Brucella* sp. INV PRT 0273 (strain 77137) according to their 16S rRNA (Figure 2). The sequence hits for each strain showed 94–99% homology with another strain of the BLAST database [21], with 0% sequence gaps in the alignment. After removing identical sequence hits, the top 10 hits with highest percentage of identity were selected for alignment and tree construction.

The Maximum Likelihood method [22] in MEGA11 placed *Stenotrophomonas* sp. INV PRT0231 in a group of environmental and clinical isolates with various *Stenotrophomonas* species, particularly with *S. maltophilia*. The strain also shared a node with *Enterobacter* sp. INV PRT0220, grouping them in the same clade. In addition, *Enterobacter* sp. INV PRT0220 was also related to species of *Enterobacter*, *Pantoea,* and *Leclercia*, which were found in both clinical and environmental sources, indicating their diverse ecological roles.

Although the three bacterial strains belong to the same phylum, Pseudomonadota, *Stenotrophomonas*, and *Enterobacter* are classified under the class Gammaproteobacteria, known for their environmental versatility and clinical significance [23,24]. On the other hand, *Brucella* belongs to the class Alphaproteobacteria, characterized by its role in various infectious diseases and ecological interactions [25].

### 3.3. Growth Curves of Selected Candidates

**Figure 3:**
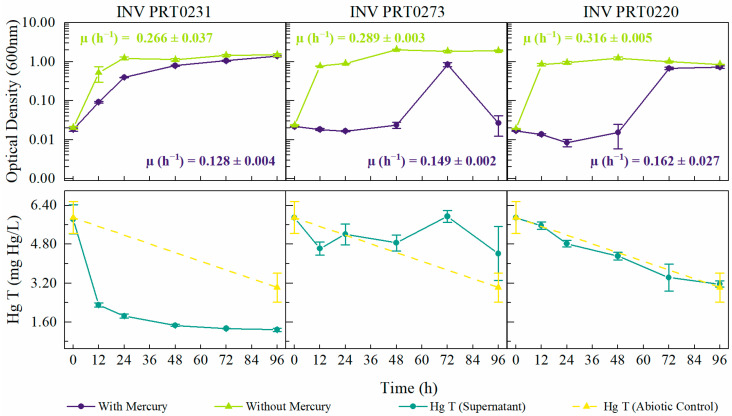
Bacterial growth curves of the three selected strains studied in this work exposed to HgCl_2_ (5.9 ± 0.7 mg Hg/L), and total Hg concentration along time. The growth curve of a control without HgCl_2_ has also been represented. INV PRT0231: *Stenotrophomonas* sp., INV PRT0273: *Brucella* sp., INV PRT0220: *Enterobacter* sp. Mean and standard deviation from three replicate samples are also shown.

The three strains identified as *Brucella* sp. INV PRT0273, *Enterobacter* sp. INV PRT0220, and *Stenotrophomonas* sp. INV PRT0231 were selected to characterize their growth curves in the presence of 5.9 ± 0.7 mg Hg/L (prepared with HgCl_2_). The lag phase was longer in all bacteria cultures compared with the growth control without Hg^2+^ (Figure 3). For the bacterium *Stenotrophomonas* sp. INV PRT0231 grown with Hg^2+^, the exponential phase was observed between 0 and 48 h, with a cell density (measured as OD_600nm_) of 0.792 ± 0.022; this value was lower compared with the OD observed in the growth control culture (1.113 ± 0.082 at 48 h), where the exponential phase occurred within the first 24 h. After 48 h, in the presence of mercury, the bacterium enters the stationary phase. The mercury reduction value at 12 h of growth was 66.0%, reaching a percentage of 85.4% (0.864 ± 0.069 mg Hg/L) after 96 h. This percentage was almost two-fold higher than the abiotic reduction observed (48.7%). Additionally, changes in the pH of the culture without mercury were observed, increasing from 7.11 ± 0.01 to 9.02 ± 0.02.

For strains INV PRT0220 and INV PRT0273, the lag phase with Hg was observed over the first 48 h of incubation, with a cell density of 0.015 ± 0.010 (25.6 ± 12.7 mg dry biomass/L) and 0.024 ± 0.004 (12.2 ± 2.5 mg dry biomass/L), respectively. From this time, the growth of INV PRT0220 increased exponentially and reached a cell density of 0.728 ± 0.054 (683.3 ± 35.6 mg dry biomass/L) at 96 h, while strain INV PRT0273 maximum growth was observed at 72 h with an OD_600nm_ of 0.843 ± 0.101 (486 ± 198.4 mg dry biomass/L). The mercury concentration in both bacterial cultures was almost equal to the abiotic control (3.0 ± 0.6 mg Hg/L), with values of 3.2 ± 0.1 mg Hg/L and 4.4 ± 1.1 mg Hg/L, respectively. These results indicate that the removal of mercury was not associated with microbial growth but was mainly due to abiotic losses, suggesting an alternative strategy for mercury tolerance other than reduction.

According to these results, the strain *Stenotrophomonas* sp. INV PRT0231 was selected for further experiments to be characterized under different environmental conditions.

### 3.4. Mercury Reduction of Stenotrophomonas sp. INV PRT0231 in Acidic Conditions

**Figure 4:**
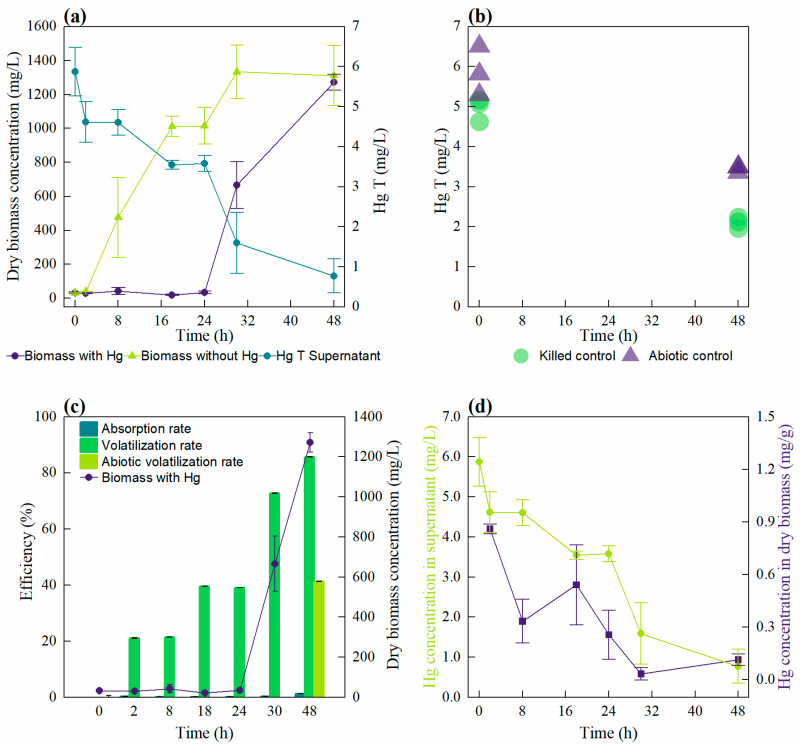
Growth and mercury removal evaluation of *Stenotrophomonas* sp. INV PRT023 at pH 6.0. (**a**) Dry biomass concentration (mg/L) of two cultures with 5.9 ± 0.6 mg Hg/L and without Hg, and total mercury concentration of the culture with Hg. (**b**) Total mercury concentration in the abiotic and killed controls. (**c**) Dry biomass concentration (dots) and % of absorption and volatilization rates from a culture grown with 5.9 ± 0.6 mg Hg/L; the volatilization rate of the abiotic control is also indicated. (**d**) Mercury concentration in dry biomass and supernatant of the culture grown with 5.9 ± 0.6 mg Hg/L. Mean and standard deviation from three replicate samples are also shown.

The mercury-resistant bacterium *Stenotrophomonas* sp. INV PRT0231 was finally selected for its ability to remove 85.3% of mercury in liquid culture at pH 7.0 after 96 h of incubation. In order to maintain mercury stability during the detoxification process and assess the optimal conditions for mercury detoxification, we subsequently investigated in detail if the strain was able to remove mercury under acidic conditions. Acidic pH favors the mercury primarily remains as Hg^2^⁺ (soluble form), facilitating its bioavailability and interaction with biomolecules [26]. Thus, we tried to characterize the growth curves of the microorganism to determine the specific growth rates (µ) in a control without HgCL_2_ and in cultures with 5.9 ± 0.6 mg Hg/L at pH 6.0. The bacterial culture growing with HgCl_2_ at pH 6.0 showed a lag phase over the first 24 h of incubation, reaching an OD_600nm_ of 0.015 ± 0.005 (34.7 ± 8.1 mg dry biomass/L). After this time, the growth increased exponentially, reaching an OD_600nm_ value of 0.41 ± 0.07 (1272 ± 48 mg dry biomass/L) after 48 h (Figure 4a). This final time showed a mercury concentration in the supernatant of 0.8 ± 0.4 mg Hg/L (Figure 4a), representing a removal rate of 86.9%, an absorption rate of 1.2%, and a volatilization rate of 85.7% (Figure 4c).

On the other hand, the abiotic reduction of mercury was similar among all control culture media evaluated at pH 6.0 and 7.0 (Figure 4b), with a percentage of reduction after 48 h of 41.3% (3.45 ± 0.08 mg Hg/L) and 48.7% (3.02 ± 0.60 mg Hg/L), respectively. The killed control supernatant showed a mercury concentration of 2.1 ± 0.1 mg Hg/L at 48 h of exposition, corresponding to 57.5% of mercury reduction. Additionally, we found a low concentration of mercury in the dry biomass and the supernatant after 48 h (Figure 4d), with values of 0.07 ± 0.02 mg Hg/L and 0.77 ± 0.42 mg Hg/L, respectively. Similarly, in the dry biomass of the killed control, we found values of 0.02 ± 0.02 mg Hg/L after 48 h of exposition.

In addition to the specific growth rates at pH 7.0 and 6.0 with and without Hg, we also calculated the removal efficiency in mg Hg∙h⁻^1^∙g biomass⁻^1^, which are summarized in Table 1. While the specific growth rates were very similar at both pH levels when the microorganism was grown without mercury (0.266 ± 0.037 h⁻^1^ at pH 7.0 and 0.376 ± 0.156 h⁻^1^ at pH 6.0), there was a significant difference in the presence of HgCl_2_. At pH 7.0, the specific growth rate dropped to 0.128 ± 0.004 h⁻^1^, with a mercury reduction efficiency of 85.4% and a removal efficiency of 1.2 mg Hg∙h⁻^1^∙g biomass⁻^1^. In contrast, at pH 6.0, the microorganism exhibited a much higher specific growth rate of 0.493 ± 0.067 h⁻^1^, a slightly higher mercury reduction of 86.9%, and a significantly increased removal efficiency of 8.4 mg Hg∙h⁻^1^∙g biomass⁻^1^. This highlights the enhanced performance of the strain at lower pH in terms of both growth and mercury removal efficiency when exposed to HgCl_2_.

### 3.5. FT-IR Analysis

**Figure 5:**
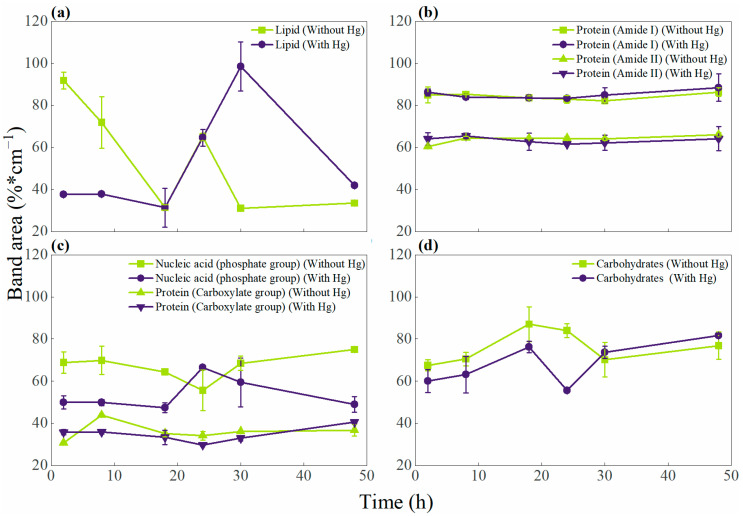
Changes in the FT-IR band area of *Stenotrophomonas* sp. INV PRT0231 biomass during its growth in cultures supplemented with Hg^2^⁺ (purple line) and cultures without Hg^2^⁺ (green line) for the distinctive functional groups present in (**a**) spectral window W1: lipids; (**b**) spectral window W2: proteins and peptides; (**c**) spectral window W3: mixed region; and (**d**) spectral window W4: Carbohydrates.

An FT-IR pattern was observed in the mid-infrared region of the electromagnetic spectrum, between 4000 and 400 cm⁻^1^, characteristic of bacterial biomass (Figure S2). These infrared spectra are divided into five spectral windows, as defined by Helm and colleagues [27], which are characteristic of bacteria and classified as follows: W1: lipid signals; W2: protein and peptide signals; W3: mixed region; W4: polysaccharide signals; and W5: fingerprint region. The functional groups (FGs) associated with these windows are listed in Table S1 of the supplementary material. Additionally, the graphs in Figure 5 show variations in the area of the transmittance bands in FG of different biomolecules over time during the growth of *Stenotrophomonas* sp. INV PRT0231, compared to conditions with and without mercury (HgCl_2_). In Figure 5a, the symmetric and asymmetric C-H stretching bands of the fatty acid chains and methyl groups—both associated with lipids—decreased during the first 18 h under both conditions. However, in the presence of mercury, a significant increase in these bands was observed around 30 h, while in the absence of mercury, the values remained low. In Figure 5b, which illustrates the changes in the FGs of proteins (Amide I and II), the band areas remained stable, showing no significant differences between samples with and without mercury, with values ranging from 60% to 80%. Figure 5c displays the bands of the phosphate FG of nucleic acids, which exhibited slight fluctuations in the first 8 h under both conditions. From 18 h onward, a marked difference in trends was noted, reaching a maximum and a minimum at 24 h in the presence and absence of mercury, respectively. Figure 5c also shows variations in the bands of the carboxylate FG (COO⁻) in proteins. The data presented in the graph without mercury showed more pronounced maximum and minimum peaks compared to the data in the graph with mercury, which maintained lower values. Finally, Figure 5d illustrates that the FG of carbohydrates increased their band areas under both conditions; however, those not exposed to mercury reached a higher maximum value than those in the presence of the metal. In summary, the results indicate that mercury exposure differentially affected the evaluated biomolecules, with more pronounced variations observed in lipids, the carboxylate group in proteins, and carbohydrates.

## 4. Discussion

The Chocó region in Colombia is characterized by high annual precipitation and hydric richness, where the Atrato, San Juan, and Baudó are the three main rivers. The environmental conditions in this region generate an ecosystem with a high biodiversity and mineral wealth, where the main economic activity is alluvial gold mining extraction in the three rivers mentioned above [28]. For gold extraction, considerable amounts of mercury are added to form an amalgam with the gold for its recovery, causing mercury concentrations to be introduced into the environment, affecting the health of the ecosystems, organisms, and human population that have access to the aquatic resources or terrestrial zones impacted [4].

Different reports indicate the presence of mercury pollution in sediment samples near gold extraction processing areas of the Atrato River, where mercury levels showed a range of total mercury values (T Hg) of 0.11−0.14 μg/g dry weight, while downstream waters showed T Hg levels between 0.07 and 0.10 μg/g dry weight [4]. Similarly, the mercury levels upstream of the San Juan River showed high concentrations of T Hg (0.174 μg/g), values that remain 20 years later in the sites where there was gold mining activity [29]. These environmental conditions could explain the mercury resistance shown by the microorganisms isolated from the localities of the Atrato and San Juan river mouths.

The high mercury concentrations in the environment may favor the presence of tolerant microorganisms, which can increase five-fold higher in the polluted environment, representing a range of 1–10% of the cultivable aerobic heterotrophic bacteria [30]. In our study, 16% of the cultivable bacteria grown in LB medium showed moderate to high mercury resistance, and according to the processing methods of the sediment samples, there was no significant difference in the number of microorganisms that showed resistance among sites. We found that the isolated bacteria were able to grow and remain metabolically active in high mercury concentrations (over 10.0 mg/mL HgCl_2_), while fungal growth was observed up to a concentration of 10.0 mg/mL HgCl_2_. These microorganisms, generally the bacteria, have evolved different mechanisms for Hg detoxification, employing enzyme-mediated transformation via the *mer* operon, functional groups in the cell wall, and extracellular polymeric substance (EPS) production, while intracellular adsorption and accumulation are the mechanisms reported for fungi [31].

Based on the results of screening with HgCl_2_, six bacteria isolated from different localities were selected to confirm their MIC values in increasing concentrations of Hg^2+^. The results revealed that three bacteria identified as *Brucella* sp. INV PRT0273, *Enterobacter* sp. INV PRT0220, and *Stenotrophomonas* sp. INV PRT0231 showed moderate to high mercury resistance levels. The evaluation of Hg^2+^ detoxification at neutral pH showed that only the bacterium identified as *Stenotrophomonas* sp. INV PRT0231, isolated from the mangrove area at the San Juan River mouth, decreased the mercury concentration in a liquid culture, while the bacteria *Brucella* sp. INV PRT0273 and *Enterobacter* sp. INV PRT0220 showed similar values to the abiotic reduction recorded in the control medium, suggesting that an alternative strategy to tolerate mercury other than reduction could be present in these microorganisms. Thus, further studies would be needed to elucidate which mechanisms they are using to tolerate this pollutant; a process related to biosorption could be a possibility. The abiotic reduction could be due to chemical reactions with amino acids or reducing compounds present in the components of the LB culture medium (tryptone and yeast extract), favored by the experimental temperature (30 °C) [26,32]

We found that the removal of mercury concentration in the liquid culture of strain INV PRT0231 was greater at acid pH, with a removal efficiency of 8.4 mg Hg·h^−1^·g biomass^−1^, a value which is almost seven-fold higher than that found in the culture medium with Hg^2+^ at pH 7 (1.2 mg Hg·h^−1^·g biomass^−1^). It has been reported that intracellular accumulation is not the most common mechanism for mercury microbial resistance [1]. Additionally, it was observed that the specific growth rate at pH 7.0 in the presence of mercury was nearly half (0.13 h⁻^1^) compared to the conditions without mercury (0.27 h⁻^1^). However, at pH 6.0, the presence or absence of Hg did not represent a significant difference in the specific growth rates observed, remaining at 0.49 h⁻^1^ and 0.38 h⁻^1^, respectively. This behavior observed at pH 6.0 is consistent with other studies, where mercury did not affect the specific growth rate of different mercury-resistant bacterial strains, although it did extend the adaptation phase [33].

Accordingly, in our study, the low amount of Hg in the biomass suggests that its removal may be related to cellular and genetic mechanisms employed by the bacterium, such as the biotransformation of mercury species (Hg^2+^ to Hg^0^) mediated by the *mer* operon. The activity of the *mer* operon is widely studied in Gram-negative bacteria and converts Hg^2^⁺ into Hg^0^ through the action of the enzyme mercury reductase (*mer*A). Hg^0^ is highly volatile and has low solubility in water, so it is rapidly removed into the atmosphere [26,34]. These may explain the high mercury removal rate in the culture, where volatilization could be the principal function employed by bacterium INV PRT0231 for detoxification supported by the interactions between functional groups detected by FT-IR, such as COO⁻, CH, and phosphates, which indicate that the strain’s biomolecules could play a role in the initial adsorption and intracellular transport of mercury before its biotransformation.

The high resistance of *Stenotrophomonas* sp. INV PRT0231 was not only observed in mercury. In a previous study, we found that this bacterium was able to grow in the presence of 350 mg/L of cadmium (Cd) and lead (Pb) and 50 mg/L of zinc (Zn) [14], indicating a high potential for removing different heavy metals. The genus *Stenotrophomonas* has been widely isolated from marine and estuarine environments polluted with hydrocarbons, pesticides, and heavy metals [35,36], and it has capabilities to resist toxic metals due to the presence of enzymes, as well as a broad spectrum of efflux pumps, strategies that provide the microorganism with the ability to degrade a wide range of compounds including pollutants [37,38].

In the phylogenetic tree, *Stenotrophomonas* sp. INV PRT0231 clusters within a clade alongside several strains of *Stenotrophomonas maltophilia*, suggesting a possible evolutionary relationship between them. Different studies have revealed the ability of *S. maltophilia* to resist heavy metals, including mercury, copper, and arsenic, as well as other metals like cadmium, zinc, and lead [39,40]. Additionally, genetic determinants for heavy metal resistance, particularly against mercury, have been reported for this species, including a transposon-derived *mer* operon (merRTPADE), known to transport and reduce Hg^2+^ into Hg^0^ [41,42]. It might be interesting to complement this analysis with studies that explore specific genes related to these resistance mechanisms to confirm whether *Stenotrophomonas* sp. INV PRT0231 shares similar resistance traits.

In the FT-IR analyses, we observed different trends in the chemical composition of the dry biomass from the culture exposed to Hg^2+^ (HgCl_2_) compared to the control without Hg. In summary, concerning lipids, the C-H stretching bands in fatty acid chains and methyl groups exhibited notable changes throughout bacterial growth, highlighting a marked difference in the trend of the bands after 24 h, where the band area increased in the presence of mercury (window W1). Additionally, we identified variations in the signals of proteins and lipopolysaccharides associated with the carboxylate group (COO⁻) in the mixed region (window W3). These interactions may be related to the mechanisms of mercury detoxification through electrostatic interactions and complex formation with the metal. Proteins associated with the cell wall can act as biosorption sites for metal ions, facilitating the active transport or passive diffusion of metals into the cytoplasm [43]. Subsequently, biotransformation involves the formation of protein–metal complexes that, after being transported across the membrane, are transformed into less toxic forms through enzymatic reactions [1]. Additionally, signals from the phosphate group, likely associated with nucleic acids (window W3) equal to extracellular DNA and membrane components, including lipopolysaccharides, were identified. After 18 h, trends between both treatments diverged, suggesting that extracellular DNA may be involved in the chelation of cations such as Hg^2^⁺ [44].

On the other hand, the transmittance bands associated with carbohydrates (window W4) showed changes in the area between 18 and 30 h, depending on the treatments. These signals indicate the secretion of EPS of anionic nature, which tend to bind non-specifically to cations like Hg^2^⁺ [1]. Extracellular polymeric substances play a crucial role in mitigating heavy metal contamination. They consist of a complex mixture of high-molecular-weight biopolymeric by-products, with exopolysaccharides being the most involved component for ion sequestration [45]. In fact, we observed the excretion of exopolysaccharides by strain INV PRT0231 when exposed to Hg^2+^. Bacterial cells may protect themselves from toxic metal ions by trapping and preventing their entry, covering their surface with a shield of EPS [46].

Microorganisms that can tolerate relatively high concentrations of heavy metals through adaptive mechanisms are of great interest due to their biotechnological applications in heavy metals removal. The use of autochthonic bacteria, such as *Stenotrophomonas* sp. INV PRT0231, enhances the effectiveness of bioremediation processes, as these microorganisms are naturally adapted to local environmental conditions [47]. This strain shows promise for mercury removal in polluted marine and estuarine ecosystems and can be developed into a bioremediation agent to detoxify mercury-contaminated wastewater.

## 5. Conclusions

This study shows the potential of microorganisms isolated from estuarine environments in the Colombian Pacific Sea to grow in the presence of mercury. Promising Gram-negative bacteria *Stenotrophomonas* sp. INV PRT0231, isolated from sediment samples pretreated with mercury chloride from the mangrove area at the San Juan River mouth, exhibited a high mercury removal rate at acidic pH (86.9%), representing a removal efficiency of 8.4 mg Hg·h^−1^·g biomass^−1^. According to our results, we suggest that the bacterium used cellular and genetic mechanisms to biotransform inorganic mercury to elemental mercury. The FT-IR analyses revealed that biomolecules and their associated functional groups also played an important role in the various mechanisms employed by the bacterium to detoxify mercury. However, further comprehensive studies are required, particularly incorporating transcriptomic analysis to investigate the expression of genes and proteins involved in mercury transport, biosorption, bioaccumulation, and biotransformation. Such investigations would offer deeper insights into the molecular interactions underlying these detoxification mechanisms, particularly concerning the functional groups that mediate these interactions.

## Figures and Tables

**Table 1:** Specific growth rate (µ), % of mercury removal, and mercury removal efficiency of *Stenotrophomonas* sp. INV PRT0231 in different culture conditions.

pH	µ (h^−1^) with Hg	µ (h^−1^) without Hg	Mercury Reduction (%)	Removal Efficiency (mg Hg∙h⁻^1^∙g Biomass^−1^)
7	0.128 ± 0.004	0.266 ± 0.037	85.4	1.2
6	0.493 ± 0.067	0.376 ± 0.156	86.9	8.4

## Data Availability

The data presented in this study are available in the Supplementary Material.

## Supplementary Materials

The following supporting information can be downloaded at: https://www.mdpi.com/article/10.3390/microorganisms12122631/s1, Figure S1: Maximal optical density at 600 nm of the six bacterial strains selected at increasing concentrations of HgCl_2_. The vertical dashed line corresponds to the Minimal Inhibitory Concentration 90 (MIC_90_). The number at the upper part of the graphs corresponds to the consecutive number of each strain from the collection of the Marine Natural History Museum of Colombia-Makuriwa located in INVEMAR. Two experimental replicates were performed; Figure S2: FT-IR analysis of *Stenotrophomonas* sp. INV PRT0231 biomass in cultures supplemented with Hg^2^⁺ and without Hg^2^⁺ after 24, 30, and 48 h; Table S1: Spectral band assignment for peaks observed in the biomass of *Stenotrophomonas* sp. INV PRT0231 and their association with the different spectral windows, adapted from [48].
