# Supplementary material for: Mercury-Resistant Bacteria Isolated from an Estuarine Ecosystem with Detoxification Potential
Source: Microorganisms. 2024 Dec 19;12(12):2631. doi: 10.3390/microorganisms12122631 (PMC11676337; doi:10.3390/microorganisms12122631)
Supplement: Supplementary file 1 [file microorganisms-12-02631-s001.zip › microorganisms-3311337-supplementary.pdf]

Supporting information: Mercury-resistant bacteria isolated from an estuarine ecosystem with detoxification potential

Marynes Quintero[1], Sol D. Zuluaga-Valencia[1], Lady Giselle Ríos-López[1], Olga Sánchez[2], Cesar A. Bernal[3], Niza Sepúlveda[4], Javier Gómez-León[1]*.

[1] Marine Bioprospecting Line, Evaluation and Use of Marine and Coastal Resources Program–VAR. Marine and Coastal Research Institute – INVEMAR. Santa Marta, Magdalena, Colombia; marynes.quintero@invemar.org.co; sol.zuluaga@invemar.org.co; lady.rios@invemar.org.co; javier.gomez@invemar.org.co

[2] Department of Genetics and Microbiology, Faculty of Biosciences, Universitat Autònoma de Barcelona, Bellaterra, Spain; olga.sanchez@uab.cat

[3] Marine Environmental Quality Laboratory Unit – LABCAM, Marine Environment Quality Program – CAM. Marine and Coastal Research Institute–INVEMAR. Santa Marta, Magdalena, Colombia; cesar.bernal@invemar.org.co

[4] Environmental Biotechnology Research Group, Faculty of Engineering, Technological University of Choco "Diego Luis Cordoba", Quibdó, Chocó, Colombia.; nizasepulveda@gmail.com

* Correspondence: javier.gomez@invemar.org.co; Tel.: +57+60+5+4328600 Ext 231

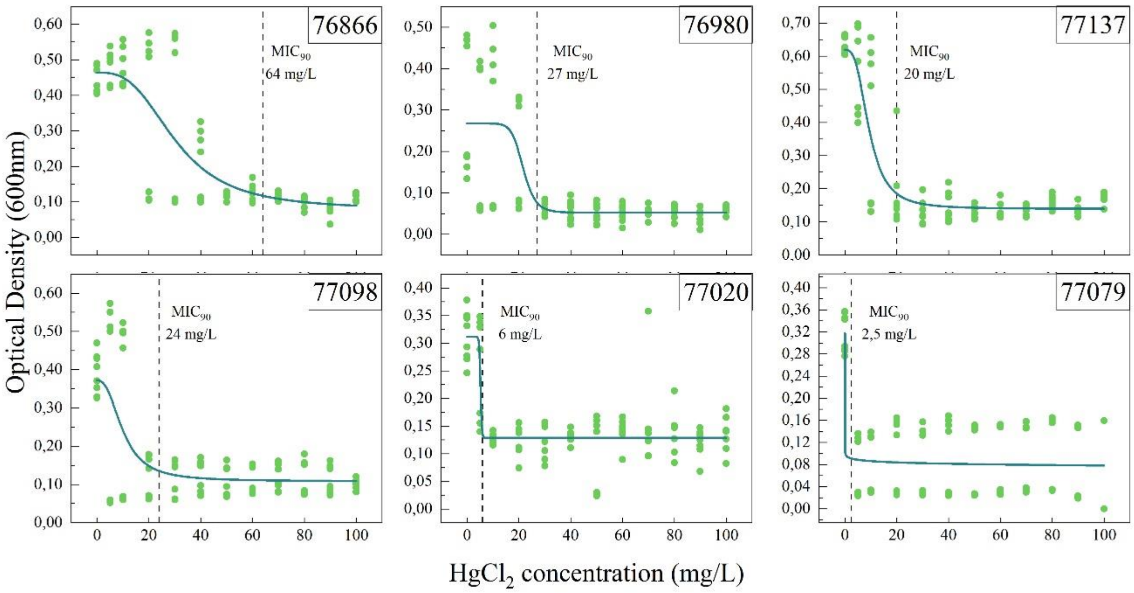

Figure S1. Maximal optical density at 600 nm of the six bacterial strains selected at increasing concentrations of $HgCl_2$. The vertical dashed line corresponds to the Minimal Inhibitory Concentration 90 (MIC_{90}). The number at the upper part of the graphs corresponds to the consecutive number of each strain from the collection of the Marine Natural History Museum of Colombia-Makuriwa located in INVEMAR. Two experimental replicates were performed.

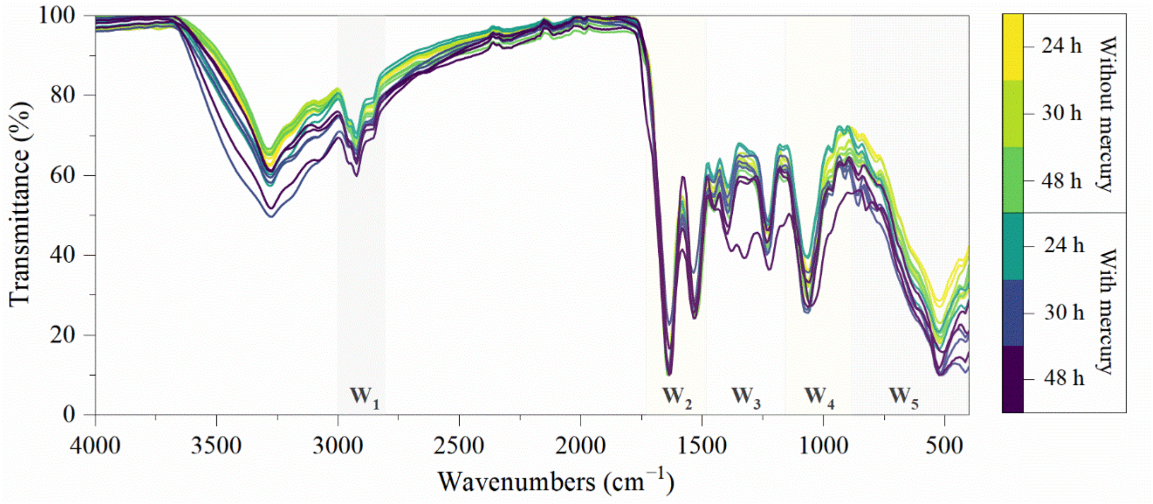

Figure S2. FT-IR analysis of *Stenotrophomonas* sp. INV PRT0231 biomass in cultures supplemented with Hg^{2+} and without Hg^{2+} after 24, 30, and 48 hours.

Table S1. Spectral band assignment for peaks observed in the biomass of *Stenotrophomonas* sp. INV PRT0231 and their association with the different spectral windows, adapted from [47].

Spectral windows	Region Spectral windows (cm^{-1})	Frequency (cm^{-1})	Assignment
**W1**	3000–2800	3000–2800	C–H vibrations of $–CH_3$ and $> CH_2$ functional groups dominated by fatty acid chains (e.g., phospholipids)
**W2**	1800 - 1500	1635,64	Amide I, >C = O str and C–N bending of protein and peptides amide
		1627–1615	>C = O str (asym) COO^{-}
		1535,34	Amide II, N–H bending, C–N str of proteins and peptides
**W3**	1500 - 1200	1450,47	C-H b of CH_2
		1400 –1410	>C = O str (sym) of COO^{-} and C–O bend from COO^{-}
		1396,46	COO^{-} sym str
		1311,59	C–O–C str of esters
		1234,44	Superimposed bands typical of different > P = O str (asym) in phosphodiesters, free phosphate, and

			monoester phosphate functional groups
			P = O str (asym) of > PO_2^- phosphodiester, functional groups of DNA/RNA polysaccharide backbone structure, phospholipids, phosphorus-containing carbohydrates
**W4**	1200 - 900	1200–900	C–OH str modes and C–O–C, C–O ring vibrations of carbohydrates (oligo, polysaccharide, and alginate), C–O–P, P–O–P in polysaccharides of cell well. P = O str (sym) of PO_2^- in nucleic acids
**W5**	900 - 700	900–800	Glycosidic linkage type “anomeric region”
		720	C–H rocking of > CH_2 methylene
		900–600	“Fingerprint region”
